# Using Lipoamidase as a Novel Probe To Interrogate the Importance of Lipoylation in Plasmodium falciparum

**DOI:** 10.1128/mBio.01872-18

**Published:** 2018-11-20

**Authors:** Hugo Jhun, Maroya S. Walters, Sean T. Prigge

**Affiliations:** a W. Harry Feinstone Department of Molecular Microbiology and Immunology, The Johns Hopkins Bloomberg School of Public Health, Baltimore, Maryland, USA; Glasgow Biomedical Research Center; University of Pittsburgh

**Keywords:** acetyl-CoA, lipoamidase, lipoate, lipoic acid, malaria, mitochondrion

## Abstract

Lipoate is a redox active cofactor that is covalently bound to key enzymes of oxidative metabolism. Plasmodium falciparum is auxotrophic for lipoate during the intraerythrocytic stages, but it is not known whether lipoate attachment to protein is required or whether attachment is required in a specific subcellular compartment of the parasite. To address these questions, we used an enzyme called lipoamidase (Lpa) as a probe of lipoate metabolism. Lpa was first described in Enterococcus faecalis, and it specifically cleaves protein-bound lipoate, inactivating enzymes requiring this cofactor. Enzymatically active Lpa could be expressed in the cytosol of P. falciparum without any effect on protein lipoylation or parasite growth. Similarly, Lpa could be expressed in the apicoplast, and although protein lipoylation was reduced, parasite growth was not inhibited. By contrast, while an inactive mutant of Lpa could be expressed in the mitochondrion, the active enzyme could not. We designed an attenuated mutant of Lpa and found that this enzyme could be expressed in the parasite mitochondrion, but only in conjunction with a chemical bypass system. These studies suggest that acetyl-CoA production and a cryptic function of the H protein are both required for parasite survival. Our study validates Lpa as a novel probe of metabolism that can be used in other systems and provides new insight into key aspects of mitochondrial metabolism that are responsible for lipoate auxotrophy in malaria parasites.

## Abstract (precis)

Lipoate is an essential cofactor for a small number of enzymes that are important for central metabolism. Malaria parasites require lipoate scavenged from the human host for growth and survival; however, it is not known why this cofactor is so important. To address this question, we designed a probe of lipoate activity based on the bacterial enzyme lipoamidase (Lpa). Expression of this probe in different subcellular locations allowed us to define the mitochondrion as the compartment housing essential lipoate metabolism. To gain further insight into the specific uses of lipoate in the mitochondrion, we designed a series of catalytically attenuated probes and employed the probes in conjunction with a chemical bypass system. These studies suggest that two lipoylated proteins are required for parasite survival. We were able to express Lpa with different catalytic abilities in different subcellular compartments and driven by different promoters, demonstrating the versatility of this tool and suggesting that it can be used as a probe of lipoate metabolism in other organisms.

## INTRODUCTION

Lipoic acid (6,8-thiooctanoic acid) is a metabolic cofactor required for the decarboxylation of alpha-ketoacids and the amino acid glycine in oxidative and one-carbon metabolism (1). *Plasmodium* spp. have four metabolic complexes that use the lipoic acid cofactor: pyruvate dehydrogenase (PDH), which resides in the apicoplast (2), and the alpha-ketoglutarate dehydrogenase (KDH), branched-chain alpha-ketoacid dehydrogenase (BCDH), and the glycine cleavage complex (GCV), all of which reside in the mitochondrion (3, 4). These complexes are generally composed of three enzymes named E1, E2, and E3, with the lipoic acid cofactor covalently bound to one or more conserved lysine residues on the E2 subunit. The GCV differs from other lipoylated complexes in that its substrate is not an alpha ketoacid but instead is the amino acid glycine, and its subunits are canonically called the T, H, P, and L proteins, of which the H protein is lipoylated and noncatalytic (5). However, there appears to be no evidence of the P protein in malaria parasites (6, 7).

The presence of lipoylated proteins in the apicoplast and mitochondrion indicates that both organelles possess functional lipoylation pathways. There are four enzymes encoded in the Plasmodium falciparum genome that are involved in lipoic acid acquisition: two lipoate biosynthesis enzymes, lipoate synthase (*Pf*LipA, PF3D7_1344600) and lipoate transferase (*Pf*LipB, PF3D7_0823600), and two enzymes with homology to lipoate ligases, designated LipL1 (PF3D7_1314600) and LipL2 (PF3D7_0923600). *Pf*LipA and *Pf*LipB functionally complement their Escherichia coli homologs and are hypothesized to be expressed in the apicoplast, showing that P. falciparum possesses a lipoate synthesis pathway (8). LipL1 and LipL2 work in concert to lipoylate the H protein and the E2 subunits of the BCDH and KDH. The two ligases also appear to have different subcellular localizations. LipL1 localizes exclusively to the mitochondrion (8). By contrast, LipL2 has been reported to partition between the apicoplast and the mitochondrion, but its distribution between these organelles is not uniform in the parasite population (9). Uptake and incorporation of ^35^S-lipoic acid by blood-stage parasites show that despite the potential presence of LipL2 in the apicoplast, only mitochondrial proteins are lipoylated by scavenged lipoate (4). Collectively, these results show that there are organelle-specific mechanisms of lipoate acquisition in P. falciparum, in which lipoate scavenging occurs exclusively in the mitochondrion, and lipoylation in the apicoplast relies on lipoate synthesis.

Growing evidence indicates that lipoate scavenging for use in the mitochondrion is likely vital to P. falciparum erythrocytic-stage parasites. Treatment of parasite cultures with lipoate analogs 8-bromooctanoate or 6,8-dichlorooctanoate resulted in decreased mitochondrial lipoylation and inhibited parasite growth (3, 4). This appears likely due to ligation of the analog to the mitochondrial substrates, which can be mitigated by excess lipoate, indicating that lipoate scavenging and attachment are important for survival. Consistent with these findings, the *Plasmodium* lipoate ligase responsible for all mitochondrial lipoylation, LipL1, appears to be refractory to deletion in both human (P. falciparum) and murine (Plasmodium berghei) malaria parasites (10, 11), suggesting that lipoylation is essential to both species of *Plasmodium*.

In this study, we exploited the partitioned nature of lipoate metabolism in P. falciparum to probe the effects of inactivating lipoylated complexes in the apicoplast and mitochondrion with an enzyme called lipoamidase. Lipoamidase (Lpa) is an enzyme first described in Enterococcus faecalis that can cleave lipoate from lipoylated proteins (12). Our work with E. coli demonstrated that Lpa can be used as a metabolic probe to deactivate lipoylated proteins, resulting in a growth defect (13); related observations have been made with a new class of lipoamidases related to the sirtuin family of proteins that appears to be conserved in human cells and bacteria (14, 15). The mycobacteriophage integrase system (16) was used to generate P. falciparum cell lines expressing Lpa constructs targeted to the cytosol, apicoplast, and mitochondrion. Expression of active Lpa in the cytosol did not affect parasite growth or lipoylation, demonstrating that no significant lipoate metabolism occurs in the cytosol. Parasites expressing active Lpa in the apicoplast showed a significant, but not total, decrease in lipoylated PDH E2; however, this change did not affect parasite growth. By contrast, we were unable to produce parasite lines expressing Lpa in the mitochondrion. We designed and tested a series of catalytically attenuated Lpa mutants and identified a severely attenuated mutant with about 1,000-fold-lower catalytic activity. This mutant Lpa could be expressed in the mitochondrion, but it demonstrated a severe growth phenotype that was partially rescued by chemical supplementation of acetate in the parasite growth medium. This parasite line displayed acetate-dependent growth and significantly reduced lipoylation of all three mitochondrial lipoylated proteins. These results demonstrate that mitochondrial lipoylation is essential during the blood stages of parasite development and provide insight into the essential roles that lipoylated proteins play in the mitochondrion.

## RESULTS

### P. falciparum can express the bacterial gene encoding Lpa.

**FIG 1:**
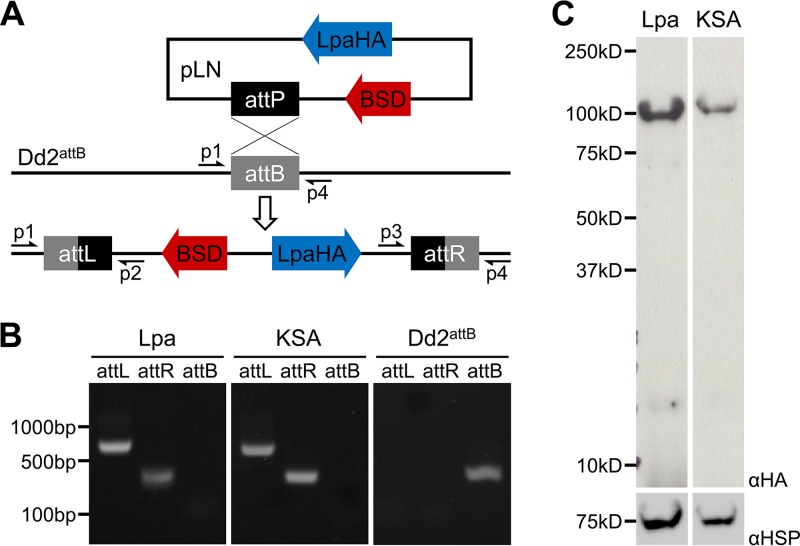
Expression of cytosolic lipoamidase in P. falciparum. (A) Model of genomic integration of the pLN plasmid expressing lipoamidase (LpaHA) and blasticidin *S*-deaminase (BSD) into the Dd2^attB^ genome. Cotransfection with the pINT plasmid provides the integrase that catalyzes the attB/attP integration. Primer pairs for amplifying the attB region (p1 and p4 [382 bp]) and the recombinant attL (p1 and p2 [770 bp]) and attR (p3 and p4 [281 bp]) regions are shown. Primers and amplicons were the same for all integration PCR experiments. (B) Genomic integration demonstrated by PCR. Both the Lpa and KSA transgenic lines demonstrate 5′ (attL) and 3′ (attR) integration and the absence of the parental attB region. The parental Dd2^attB^ line serves as a control. (C) Western blotting to confirm lipoamidase expression. Antibodies specific for the HA tag (αHA) were used to demonstrate expression of both the Lpa and KSA constructs in lysates of the transgenic parasites. Antiserum specific for *Pf*HSP70 (αHSP) was used as a loading control. There was no statistically significant difference in expression between the Lpa and KSA constructs (two-tailed Student’s *t* test, *n* = 4). kD, kilodaltons.

Lpa has been used *in vivo* as an inducible probe of alpha-ketoacid dehydrogenase inactivation in E. coli (13), and the suitability of Lpa as an *in vivo* probe hinges on the ability of the organism under study to express the bacterial enzyme in an active form. To evaluate whether P. falciparum can express Lpa, we used mycobacteriophage integrase-mediated recombination to generate parasite lines containing an integrated copy of the gene (16, 17) (Fig. 1A) encoding either active Lpa or Lpa inactivated by mutations of active site residues K159A and S259A (KSA) (13). The inactive, KSA variant was transfected in parallel experiments with the active lipoamidase to control for any molecular or growth phenotypes caused by expression of the protein itself. Lpa constructs were tagged at the C terminus with a hemagglutinin (HA) epitope tag. This generated the cell lines Lpa and KSA. Plasmid integration at the *attB* locus was confirmed by PCR (Fig. 1B).

**FIG 2:**
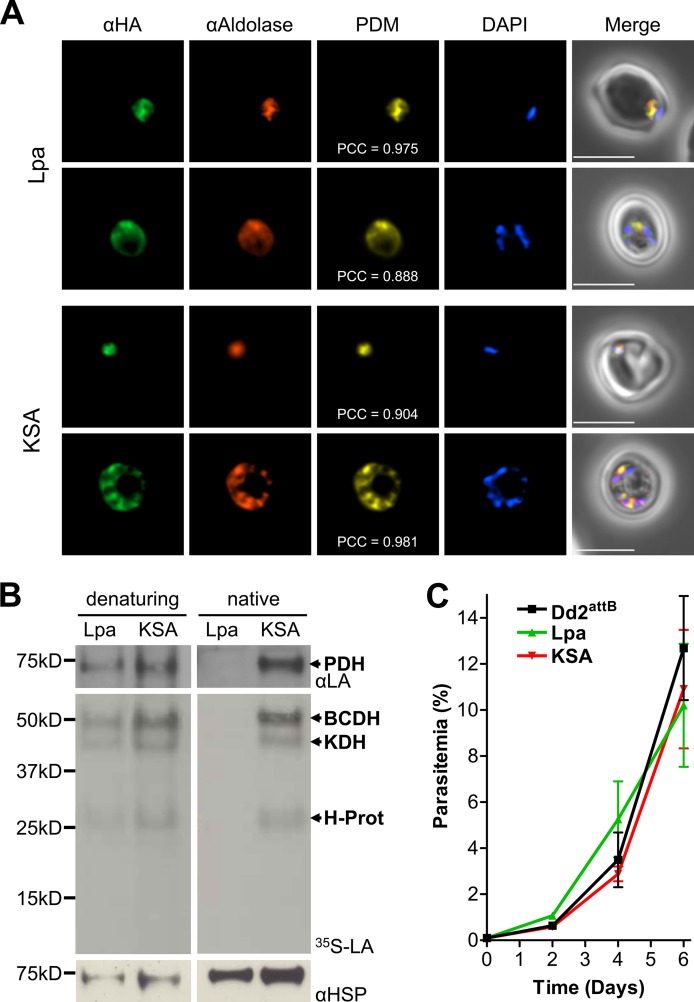
Localization, activity, and growth phenotype of cytosolic lipoamidase constructs. (A) Lpa and KSA are expressed in the cytosol. Immunofluorescence analysis (IFA) of blood-stage Lpa (top panels) and KSA (bottom panels) parasite lines showing colocalization of the cytosolic marker αAldolase and the lipoamidase constructs, labeled with an antibody specific for the hemagglutinin tag (αHA). Product of the differences from the mean (PDM) images and Pearson correlation coefficients (PCC) are shown. Mean PCC values for Lpa and KSA were 0.971 (standard error of the mean [SEM] = 0.013) and 0.944 (SEM = 0.009). Student’s *t* test of PCC values demonstrates no statistical difference between the Lpa and KSA lines. The nuclei are labeled with DAPI. Bars = 5 μm. (B) Lipoamidase is expressed in an enzymatically active form. In these multipanel figures, antilipoate (αLA) Western blot was used to identify lipoylated apicoplast PDH; autoradiograph of parasites treated with ^35^S-lipoate (^35^S-LA) was used to detect lipoylation of BCDH, KDH, and H protein; and anti-*Pf*Hsp70 (αHSP) was used as a loading control. The left panels show results for parasites expressing Lpa and KSA that were lysed in SDS-PAGE loading buffer immediately prior to analysis, showing comparable protein lipoylation between both parasite lines. Under nondenaturing (native) conditions (lysis in RIPA buffer, followed by a 15-min incubation at 30°C), cytosolic lipoamidase has access to organellar proteins and rapidly cleaves lipoate from all parasite proteins. (C) Expression of either active or inactive lipoamidase imparts no growth phenotype. The growth of Lpa (green) and KSA (red) parasite lines was monitored by microscopy at 2-day intervals to demonstrate that these lines does not differ significantly from the parental Dd2^attB^ (black) parasite line (one-way ANOVA, followed by Bonferroni’s correction). Error bars represent the SEM for three biological replicates.

Expression of Lpa was determined by SDS-PAGE fractionation of whole-cell lysates, followed by Western blotting with monoclonal antibodies specific for HA. In both cell lines, a single HA-tagged protein of approximately 100 kDa is present (Fig. 1C) at roughly equivalent levels. Untagged Lpa has previously been reported to migrate at 97 kDa, despite a predicted molecular weight of 79 kDa (12); therefore, the bands identified by anti-HA Western blot are consistent with the empirical size of Lpa. In the absence of N-terminal leader sequences, Lpa is expected to localize to the cytosol. The results of an immunofluorescence assay (IFA) demonstrate that the signal from HA-tagged lipoamidase occupies most of the parasite cell and is coincident with aldolase staining, but exclusive of the nucleus (Fig. 2A). Taken together, these results show that Lpa and the catalytically dead KSA mutant can be expressed in the cytosol of P. falciparum parasites.

### Lpa expressed in the cytosol is active and does not inhibit parasite growth.

Lipoylated proteins and the enzymes involved in lipoylation have been localized exclusively to the apicoplast and mitochondrion, so expression of active lipoamidase in the cytosol was not expected to perturb protein lipoylation. To test this hypothesis, we compared protein lipoylation in Lpa and KSA parasites by assaying apicoplast lipoylation using Western blotting with an antibody recognizing lipoylated proteins and assaying mitochondrial lipoylation by using autoradiography with cultures supplemented with ^35^S-radiolabeled lipoate. These two methods were used because parasites use exogenous lipoate to lipoylate mitochondrial proteins, while the apicoplast proteins are lipoylated through a biosynthetic pathway (4). The use of radiolabeled lipoate also allows for reliable detection of the mitochondrial proteins, especially the H protein, which is difficult to detect by Western blotting (3). When cell pellets were lysed under denaturing conditions, we found that both parasite lines showed similar levels of lipoylation relative to the *Pf*HSP70 loading control (Fig. 2B, left panels), indicating that lipoylation was not affected by Lpa expression. We then assayed Lpa activity by lysing identical samples under nondenaturing conditions, followed by incubation at 30°C for 15 min. In lysates containing active Lpa, no lipoylation was detected after incubation. It is possible that cell lysis allowed cytosolic proteases to degrade the lipoylated proteins; however, protein lipoylation was maintained in lysates of the catalytically dead KSA mutant (Fig. 2B, right panels). Thus, any proteolysis would have to be triggered by Lpa and not the KSA mutant. The more likely explanation is that Lpa is expressed by P. falciparum in an active form and that Lpa can recognize and cleave lipoate from all four P. falciparum lipoylated proteins if given access.

We then compared the growth of Lpa and KSA parasite lines to growth of the parental line to determine whether expression of the active enzyme resulted in a growth phenotype. Expression of either Lpa or KSA did not result in discernible differences in growth compared to the parental line (Fig. 2C). Coupling this observation to the unchanged lipoylation state of the Lpa-expressing line suggests that there is no lipoylated protein in the cytosol that would be important for parasite growth, nor is there off-target, deleterious activity by Lpa in the cytosol.

### Reduced lipoylation of the apicoplast pyruvate dehydrogenase does not impair parasite growth.

**FIG 3:**
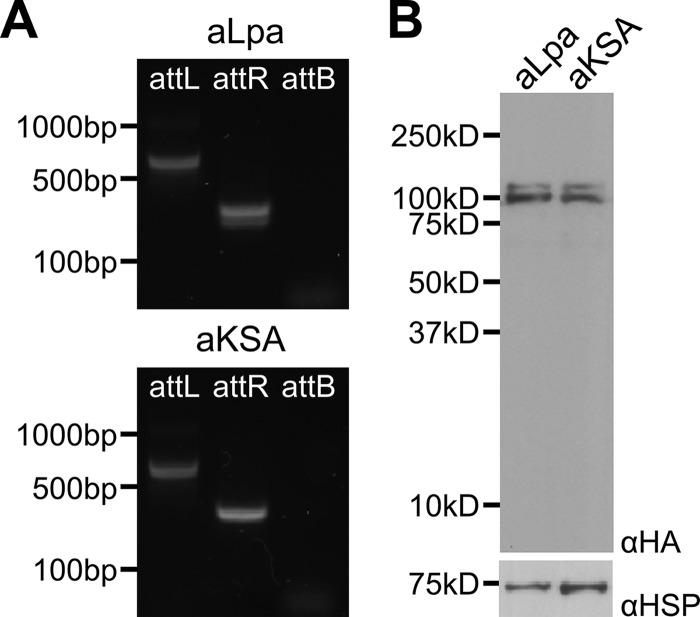
Expression of apicoplast lipoamidase in P. falciparum. (A) Genomic integration demonstrated by PCR. Both the aLpa (expressing ACP_55_Lpa) and aKSA (expressing ACP_55_KSA) transgenic lines demonstrate 5′ (attL) and 3′ (attR) integration and the absence of the parental attB region. (B) Western blotting to confirm apicoplast-targeted lipoamidase expression. Antibodies specific for the HA tag (αHA) were used to demonstrate lipoamidase expression in both the aLpa and aKSA transgenic parasites lines. A protein doublet was observed in both lines, with the lower band presumably formed after cleavage of the N-terminal apicoplast-targeting peptide. There was no statistically significant difference between aLpa and aKSA expression (two-tailed Student’s *t* test, *n* = 4). Anti-*Pf*Hsp70 (αHSP) was used as a loading control.

**FIG 4:**
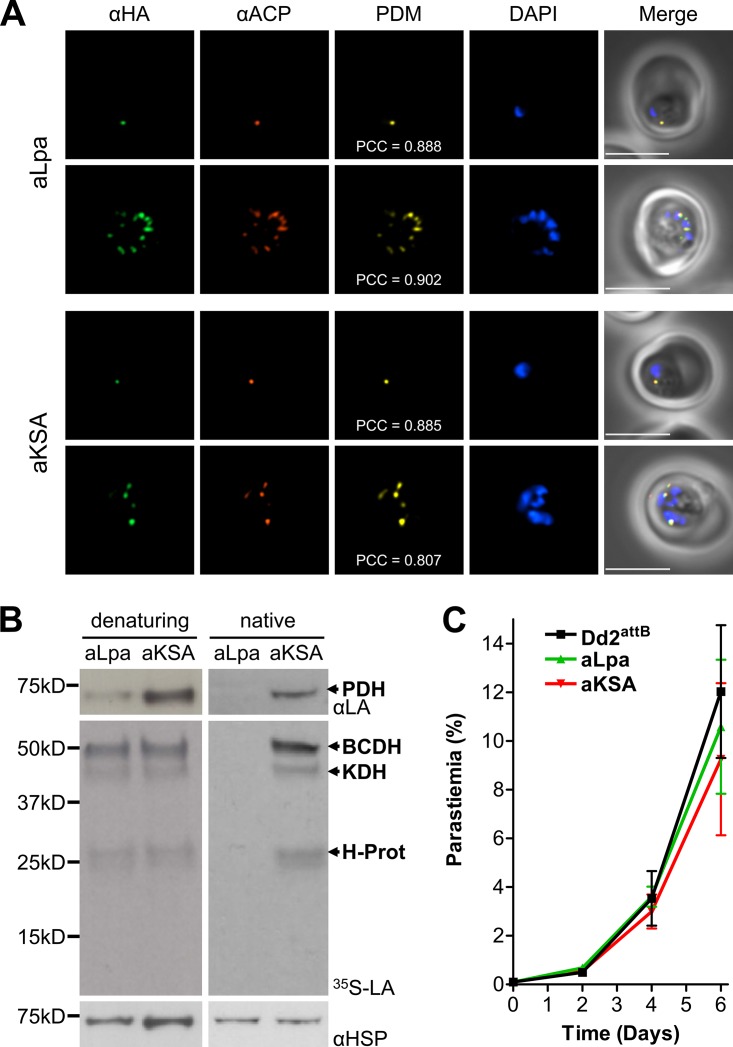
Localization, activity, and growth phenotype of apicoplast lipoamidase constructs. (A) ACP_55_Lpa and ACP_55_KSA are expressed in the apicoplast in aLpa and aKSA parasite lines. Immunofluorescence analysis (IFA) of blood-stage aLpa (top panels) and aKSA (bottom panels) parasite lines showing colocalization of the apicoplast resident acyl carrier protein (αACP) and the lipoamidase constructs, labeled with an antibody specific for the hemagglutinin tag (αHA). Product of the differences from the mean (PDM) images and Pearson correlation coefficients (PCC) are shown. Mean PCC values for aLpa and aKSA were 0.807 (SEM = 0.050) and 0.872 (SEM = 0.016), with no statistical difference between the two lines (two-tailed Student’s *t* test). The nuclei are labeled with DAPI. Bars = 5 μm. (B) Apicoplast-targeted lipoamidase can significantly reduce apicoplast-specific lipoylation. Western blot and autoradiograph panels are similar to those shown in Fig. 2B. The left-hand panels show that PDH lipoylation is significantly reduced in aLpa, but not aKSA, parasites (mean lipoylation of 38% compared to aKSA, SEM of 6.7%), while there is no difference in the lipoylation of the mitochondrial proteins. Under nondenaturing (native) conditions (lysis in RIPA buffer, followed by a 15-min incubation at 30°C), apicoplast Lpa rapidly cleaves lipoate from all parasite proteins (right panels). (C) Expression of either active or inactive lipoamidase in the apicoplast imparts no growth phenotype. The growth of aLpa (green) and aKSA (red) parasite lines was monitored by microscopy at 2-day intervals to demonstrate that these lines do not differ significantly from the parental Dd2^attB^ (black) parasite line (one-way ANOVA, followed by Bonferroni’s correction). Error bars represent the SEM for three biological replicates.

In order to determine whether lipoylated PDH is essential for the growth of blood-stage P. falciparum, we sought to express Lpa in the apicoplast. To direct Lpa to the apicoplast, the first 55 amino acids of the acyl carrier protein (ACP_55_), which was previously shown to direct GFP to the apicoplast (18), was appended to the Lpa and KSA constructs. Initial attempts to express ACP_55_Lpa (aLpa) and ACP_55_KSA (aKSA) using the strong calmodulin promoter were unsuccessful, perhaps because overexpression of apicoplast proteins by this promoter is poorly tolerated, a phenomenon that we have observed before (19). Switching to the lower-strength RL2 (ribosomal L2 protein) promoter (20) allowed us to generate parasite lines for both constructs. Integration was confirmed by PCR (Fig. 3A), and anti-HA Western blot analysis of whole-cell lysates from both parasite lines recognized a doublet characteristic of apicoplast-localized proteins (21) in which the lower band corresponds to the expected 100-kDa size of mature Lpa after import into the apicoplast (Fig. 3B). As in the cytosolic constructs, Western blotting suggests relatively equivalent expression of the two constructs. To determine the subcellular localization of the lipoamidase proteins, we performed IFA on fixed aLpa and aKSA parasites. The signal from anti-HA colocalized with the apicoplast marker anti-ACP (Fig. 4A), demonstrating that both aLpa and aKSA localize primarily to the apicoplast.

The effect of aLpa expression on lipoylation was determined by Western blotting and autoradiography. Western blotting revealed that PDH lipoylation was reduced in aLpa compared to aKSA parasites, although some lipoylation persisted (Fig. 4B, top left panel). Autoradiography of parasites cultured in the presence of ^35^S-lipoate showed that mitochondrial lipoylation was largely unaffected (Fig. 4B, middle left panel). We were curious whether low activity of aLpa [due to improper protein folding, modification of the N terminus, or some other factor(s)] was responsible for the persistence of some lipoylated PDH. To investigate this, we analyzed the activity of aLpa in parasite extracts and found that aLpa rapidly reduced lipoylation of all four parasite proteins to below the limit of detection (Fig. 4B, right panels). This result shows that aLpa is capable of completely delipoylating PDH, but in the apicoplast, some factors prevent it from doing so efficiently, such as the environmental conditions or robust lipoylation activity by the lipoate synthesis machinery. Ultimately, reduction of lipoylation in the apicoplast appears to have little effect on the parasite, since expression of aLpa does not impart a growth phenotype compared to aKSA expression or the parental line (Fig. 4C).

### Lipoamidase is toxic when expressed in the mitochondrion.

In order to target lipoamidase to the mitochondrion, the cytosolic constructs were again modified, this time to contain an N-terminal mitochondrial targeting motif using the first 33 amino acids from LipL1 (L1_33_). In repeated transfections, we were able to generate parasite lines expressing mitochondrially targeted L1_33_KSA (mKSA); however, multiple attempts at expressing L1_33_Lpa (mLpa) failed (Table 1). These results are consistent with the hypothesis that one or more of the lipoylated proteins found in the mitochondrion are essential for parasite growth. We attempted to demonstrate this phenomenon by using a conditional destabilization approach and a chemical bypass approach. In the first approach, we used the destabilization domain (DD) developed by Armstrong and Goldberg (22) to generate parasite lines expressing a destabilized lipoamidase. We were not able to generate parasites expressing lipoamidase with either an N-terminal or C-terminal DD (data not shown).

**TABLE 1:** Summary of attempted parasite transfections

Localization	Construct	No. of transfections attempted	No. of lines with successful expression
Cytosol	Lpa (Lpa)	2	2
	KSA (KSA)	2	2

Apicoplast	ACP_55_Lpa (aLpa)	2	2
	ACP_55_KSA (aKSA)	2	2

Mitochondrion	L1_33_Lpa (mLpa) alone	4	0
	L1_33_Lpa (mLpa) + acetate	1	0
	L1_33_KSA (mKSA)	4	4
	L1_33_S236A (mS236A)	2	0
	L1_33_S236A (mS236A) + acetate	3	2

We then attempted to metabolically bypass lipoamidase toxicity. Lipoamidase expression in *E. coli* inactivates the lipoate-dependent PDH and KDH, significantly reducing the production of acetyl-CoA and succinyl-CoA, respectively (13). Acetate and succinate supplementation has been described as a means of bypassing KDH and PDH in E. coli (12), suggesting that a similar supplementation strategy may work in *Plasmodium*. Metabolic analysis of P. falciparum supplemented with radiolabeled acetate demonstrated that parasites can convert exogenous acetate into acetyl-CoA (23), and 5 mM acetate was shown to restore the growth of P. berghei parasites harboring a deletion of the BCDH E1α subunit (24). However, even with chemical supplementation (5 mM acetate), we were still not able to generate parasites expressing mLpa (Table 1).

### Lipoylated proteins in the mitochondrion are essential for parasite survival.

Cell-free activity assays, such as that shown in Fig. 2B demonstrate that lipoamidase can cleave lipoate from all parasite proteins in a surprisingly short period of time. We hypothesized that mLpa activity may be too high to effectively control with the conditional tools available for P. falciparum genetics, all of which have limited dynamic ranges. We considered using low-strength promoters to drive mLpa expression; however, this approach makes it difficult to identify the expressed protein and confirm its localization. Instead, we attempted to attenuate the activity of lipoamidase with a series of active site mutations. We made a homology model of the E. faecalis lipoamidase based on the structure of a hydrolase (PDB code 3A2Q) (25) with 27% sequence identity and designed active site mutations to attenuate lipoamidase activity. Point mutations were designed to interfere with hydrogen bonds orienting the catalytic lysine K159 (S236A, S236C, and S236G), to disrupt stabilization of the amide carbonyl (A256G and Y375F), and to interfere with the hypothetical position of the lipoate dithiolan ring (W210F). Quantification of lipoamidase activity using an E. coli bioassay identified S236A as the most attenuated mutant with an approximately 1,000-fold decrease in catalytic activity (see Fig. S1 in the supplemental material). This variant was then cloned into the same transfection vector as mLpa and named mS236A.

10.1128/mBio.01872-18.1 FIG S1 Delipoylation assay showing that LpaS236A is about 1,000 times less active compared to wild-type lipoamidase. The active Lpa, inactive KSA, and mutant S236A variants of lipoamidase were expressed in lipoylation-deficient E. coli and prepared as cell lysates. The lysates were analyzed by anti-HA Western blotting and then diluted to normalize the amount of lipoamidase protein in each lysate (set at an arbitrary value of 1). Lipoylated H protein from Bacillus subtilis was added to each lysate to serve as the substrate in a 30-min cell-free delipoylation assay. As expected, adding an equivalent volume of buffer (lane 1) produced a signal similar to the KSA mutant (lane 2), demonstrating that this mutant does not have lipoamidase activity. Serial 10-fold dilutions of Lpa and S236A show that there is roughly a 1,000-fold difference in the lipoamidase activity of Lpa compared to S236A. Download FIG S1, TIF file, 1.7 MB. Copyright © 2018 Jhun et al. 2018 Jhun et al. This content is distributed under the terms of the Creative Commons Attribution 4.0 International license.

**FIG 5:**
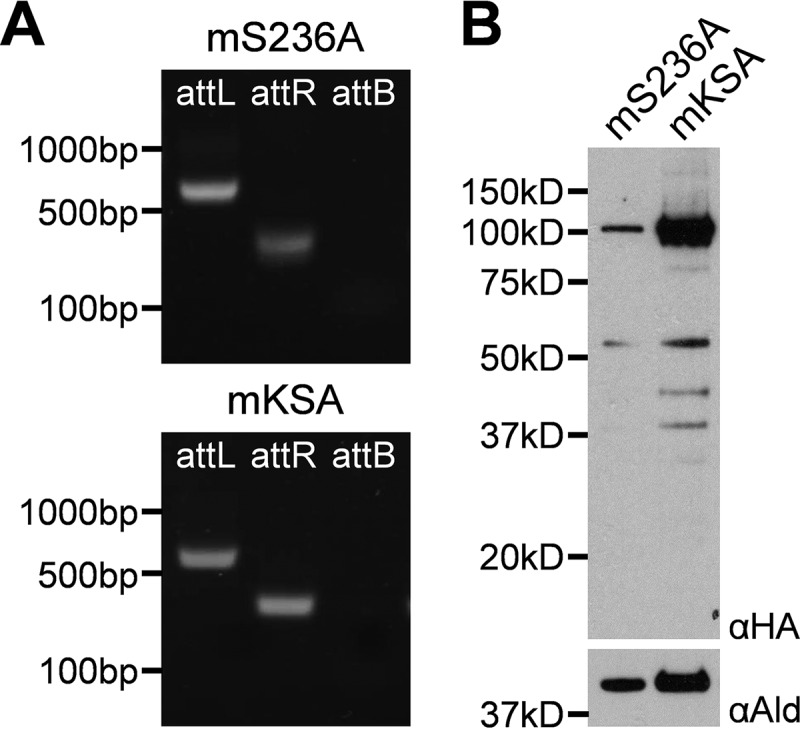
Expression of mitochondrial lipoamidase in P. falciparum. (A) Genomic integration demonstrated by PCR. Both the mS236A (expressing L1_33_S236A) and mKSA (expressing L1_33_KSA) transgenic lines demonstrate 5′ (attL) and 3′ (attR) integration and the absence of the parental attB region. (B) Western blotting to confirm mitochondrial targeted lipoamidase expression. Antibodies specific for the HA tag (αHA) were used to demonstrate lipoamidase expression in both the mS236A and mKSA transgenic parasites lines. Signal from the mS236A line shows lower expression compared to mKSA parasites, as well as possible degradation products of lower molecular weight; however, this difference was not found to be statistically significant (*n* = 4). *Pf*Aldolase (αAld) was used as the loading control.

**FIG 6:**
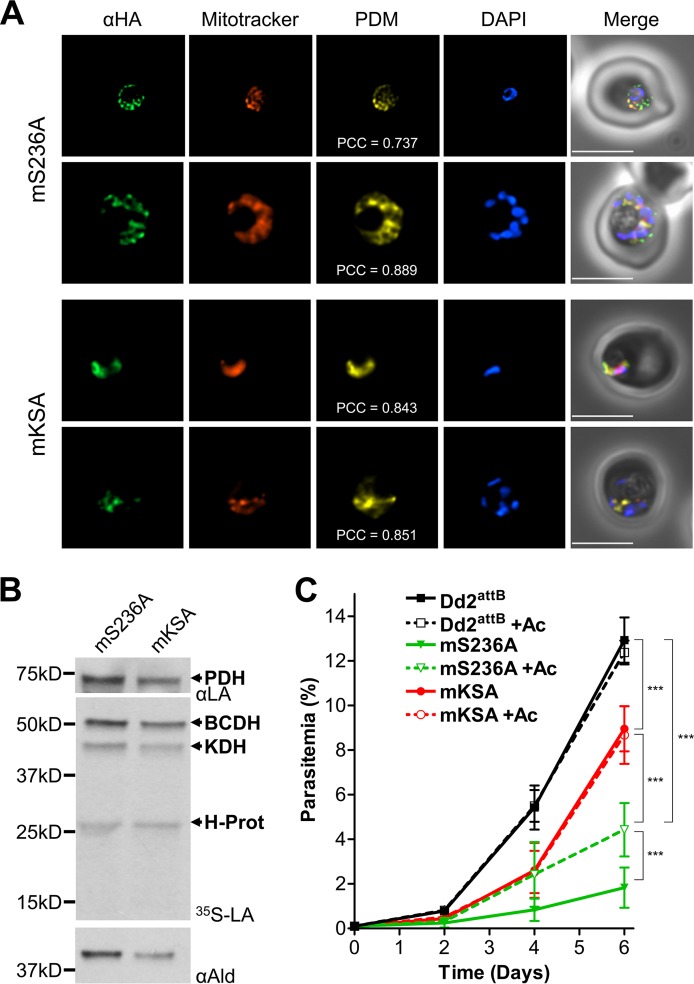
Localization, activity, and growth phenotype of mitochondrial lipoamidase constructs. (A) L1_33_S236A and L1_33_KSA are expressed in the mitochondrion in mS236A and mKSA parasite lines. Immunofluorescence analysis (IFA) of blood-stage mS236A (top panels) and mKSA (bottom panels) parasite lines showing colocalization of MitoTracker and the lipoamidase constructs, labeled with an antibody specific for the hemagglutinin tag (αHA). Product of the differences from the mean (PDM) images and Pearson correlation coefficients (PCC) are shown. Mean PCC values for mS236A and mKSA were 0.811 (SEM = 0.026) and 0.727 (SEM = 0.072) with no statistical difference between the two lines (two-tailed Student’s *t* test). The nuclei are labeled with DAPI. Bars = 5 μm. (B) Mitochondrial lipoamidase can efficiently cleave lipoate from mitochondrial substrates. Western blot and autoradiograph panels similar to those shown in Fig. 2B are shown for mS236A and mKSA parasites lysed under denaturing conditions. Lipoylation of the three mitochondrial proteins (BCDH, KDH, and H protein) are reduced in the mS236A line (by an average of 29% for all mitochondrial proteins) compared to the mKSA line. The apicoplast resident PDH E2 subunit retains comparable signal between the mS236A line and the mKSA line. (C) Mitochondrial expression of active lipoamidase imparts a growth phenotype. The mS236A line (green solid line) grows very poorly compared to the parental Dd2^attB^ parasites (black solid line), while a modest defect appears in the mKSA line (red solid line). The slow growth of the mS236A line is partially rescued by supplementation with acetate (Ac) (green dashed line), while acetate supplementation has no effect on the mKSA parasites (red dashed line) or the parental line (black dashed line) (two-way ANOVA, followed by Bonferroni’s correction; ***, *P* < 0.001). Error bars represent SEM for biological triplicates.

The mS236A lipoamidase variant was successfully expressed under supplementation with 5 mM acetate. As with the other constructs, PCR for plasmid integration and Western blotting were used to confirm expression (Fig. 5). Unlike the gene products targeted to the cytosol and the apicoplast, there was a marked difference in protein levels in the mitochondrion as observed by Western blotting. The parasite appears to have significantly decreased levels of the mS236A construct compared to mKSA, even though they are driven by the same regulatory elements. However, although we consistently observed decreased mS236A expression compared to mKSA expression, this difference was not statistically significant. In addition, there appears to be some degradation of both gene products, unlike what was demonstrated for lipoamidase expressed in the cytosol or apicoplast. Immunofluorescence was used to demonstrate mitochondrial localization using MitoTracker Red. Pearson correlation coefficients (PCC values) and product of the differences from the mean (PDM) images show that lipoamidase is successfully expressed in the mitochondrion, but there remains some minor amount of protein that is outside the mitochondrion (Fig. 6A). This holds true for mKSA expression as well, suggesting that this phenomenon is likely an artifact of the overexpression system rather than a specific effect caused by lipoamidase activity.

Western blotting revealed that the apicoplast PDH remained lipoylated in both the mS236A and mKSA parasite lines, consistent with these constructs having no impact on apicoplast protein lipoylation. By contrast, there was a significant reduction in lipoylation of all three lipoate-dependent proteins found in the mitochondrion compared to either the loading control or apicoplast PDH lipoylation (Fig. 6B). Incubating lysates under nondenaturing conditions confirms that the mS236A variant can delipoylate all lipoylated substrates, but not as efficiently as wild-type lipoamidase (Fig. S3). These results show that the attenuated mS236A lipoamidase mutant is sufficiently active to reduce but not abolish the lipoylation of mitochondrial proteins. Parasite growth curves show that the mKSA line demonstrates a minor growth defect compared to the parental line, a difference that may be due to the overexpression of mKSA by the strong calmodulin promoter. Importantly, acetate supplementation does not affect the growth of either the parental or mKSA parasite lines (Fig. 6C). The growth of the mS236A parasite line, however, is significantly improved with acetate supplementation, and parasites without acetate supplementation grow approximately 65% poorer than parasites with supplementation (Fig. S4). These results suggest that parasites are able to survive with what little lipoylation remains in the mitochondrion and can exploit acetate-generated acetyl-CoA for improved growth, but not sufficiently enough to restore growth to that of parasites expressing mKSA, or to wild-type levels. Taken together, these results demonstrate that lipoamidase cleaves lipoate from all three mitochondrial proteins, imparting a severe growth phenotype that is only partially rescued with acetate supplementation.

## DISCUSSION

**FIG 7:**
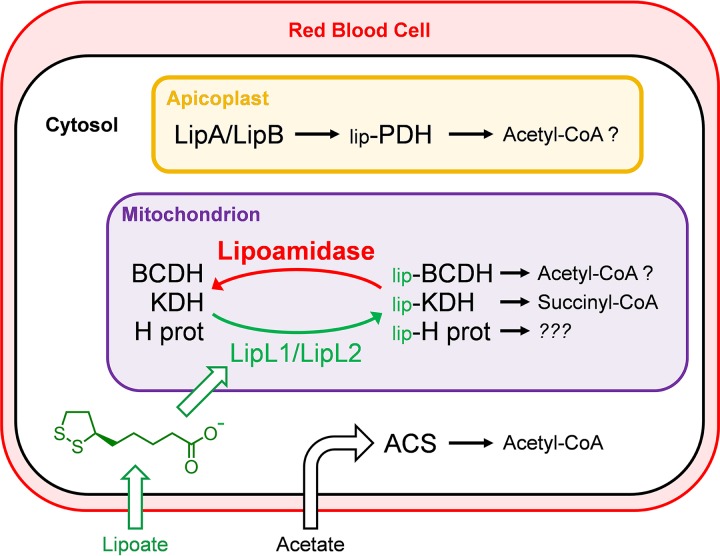
Model of parasite lipoylation and lipoamidase activity. Lipoylation of PDH in the apicoplast by LipA and LipB involves lipoate synthesis and is independent of lipoate scavenging. The normal route of lipoate scavenging and attachment by LipL1 and LipL2 is shown in green. Expression of lipoamidase (red) results in the delipoylation of the mitochondrial proteins BCDH, KDH, and the H protein. Inactivation of these proteins may reduce the production of several metabolites, including acetyl-CoA. These effects may be partially reversed by the conversion of acetate into acetyl-CoA by cytosolic ACS (acetyl-CoA synthase).

Lipoic acid is a metabolic cofactor that is acquired in an unusual manner by the parasite: although it is synthesized in the apicoplast, it is scavenged from the host for use in the mitochondrion. Experiments with lipoate analogs indicated that lipoate scavenging is essential for parasite growth (3, 4), and the results described in this paper show that scavenged lipoate must be attached to mitochondrial proteins. This conclusion is highlighted by the correlation between parasite growth and the enzymatic activity of lipoamidase expressed in the mitochondrion. Parasites grow reasonably well when expressing inactive mKSA, poorly when expressing attenuated mS236A, and do not seem to be able to survive with the fully active mLpa enzyme expressed in the mitochondrion. Parasites expressing either mS236A or mKSA are equally capable in taking up and focally accumulating MitoTracker Red CMXRos, a phenomenon requiring a functional mitochondrion to generate an electrochemical gradient (26), suggesting that this growth phenotype is not due to bulk mitochondrial failure. Lipoamidase activity (red in Fig. 7) interferes with the lipoylation of mitochondrial proteins (green in Fig. 7). A key question is which of the three lipoate-dependent proteins is essential for parasite growth and survival?

The effect of acetate supplementation provides an important insight into this question. Acetate only enhances the growth of the mS236A parasite line, suggesting that it is compensating for the inactivation of one of the mitochondrial proteins. The best candidate is the BCDH. This enzyme complex was named based on its homology to branched-chain amino acid dehydrogenases, but accumulating evidence suggests that it is more likely a mitochondrial pyruvate dehydrogenase (PDH). Stable leucine isotope labeling experiments suggest that blood-stage P. falciparum parasites do not catabolize branched-chain amino acids (23), and similar experiments in P. berghei demonstrate no incorporation of leucine into acetyl-CoA or TCA intermediates, also suggesting that there is no catabolism of branched-chain amino acids (24). Consistent with these conclusions, the genomes of malaria parasites do not appear to encode all of the enzymes necessary to metabolize branched-chain amino acids. On the other hand, parasites do have a pyruvate dehydrogenase activity that is responsible for the incorporation of carbon from glucose into the citric acid cycle (27). Knockout of the apicoplast resident PDH did not affect parasite growth or acetyl-CoA levels (23), making the case that the apicoplast PDH is not the enzyme chiefly responsible for the conversion of glucose-derived pyruvate to acetyl-CoA. Knockout of the E1 subunit of the BCDH in P. berghei, however, results in a severe growth phenotype that is partially rescued by acetate supplementation (24). The authors conclude that this rescue is likely mediated by an acetyl-CoA synthetase (ACS) that can convert acetate to acetyl-CoA (24). Our results are closely aligned with these observations: by reducing mitochondrial lipoylation with mS236A, we may be reducing acetyl-CoA generation to the point where the parasites must rely on acetate supplementation and the cytosolic ACS to generate acetyl-CoA for optimal growth (Fig. 7). Thus, lipoate scavenging may be essential for acetyl-CoA production in malaria parasites.

In addition to acetate supplementation, we also tried to see whether lipoate could rescue the growth of mS236A parasites. Supplementation with 2 μM lipoate can rescue the toxicity of the lipoate analog 8-bromooctanoate (4), and this seemed an appropriate concentration to use, since higher concentrations (greater than 5 μM) begin to affect parasite growth. Lipoate supplementation, however, did not result in a significant growth enhancement for parasites expressing mS236A. These parasites probably do not lack lipoate since lipoamidase activity generates free lipoate. It may be more a question of the kinetic equilibrium between mS236A lipoamidase activity and the rate of lipoate ligation. Supplementation with low concentrations of lipoate must not shift the activity of lipoate ligation enough to affect parasite growth.

Reducing the lipoylation of KDH may not affect the growth of blood-stage parasites. Knockout studies show that the E1 subunit of P. falciparum KDH can be deleted without affecting blood-stage parasite growth (28). Metabolite analysis of this deletion line compared to wild-type parasites confirms that KDH catalyzes the conversion of alpha-ketoglutarate to succinyl-CoA as a component of the TCA cycle (28). On the basis of these results, we would predict that lipoamidase inactivation of the E2 subunit of the KDH complex would not affect blood-stage parasite growth. However, the KDH E1 subunit is, at least *in vitro*, able to decarboxylate both alpha-ketoglutarate and pyruvate at reasonably comparable rates (29). Therefore, both the BCDH and the KDH may be contributing to the acetyl-CoA pool under different circumstances. In our experiments, lipoamidase activity affects BCDH and KDH lipoylation (Fig. 6B), reducing the ability of either enzyme to generate acetyl-CoA.

Expression of the inactive mKSA mutant in the mitochondrion affects parasite growth (Fig. 6C). The fact that acetate supplementation does not appear to rescue growth suggests that this phenomenon is independent of acetyl-CoA generation. It is possible that this is simply a consequence of overexpression of an exogenous gene interfering with normal protein trafficking or proteostasis in the parasite mitochondrion. This phenomenon appeared to be the case for the apicoplast as well, since expression of aKSA was successful only after switching to the lower-strength RL2 promoter. Acetate rescue is insufficient to fully rescue growth inhibition in parasites expressing mitochondrial mS236A, suggesting that some other process is being affected by lipoamidase activity.

The H protein may have an essential activity that is not bypassed by acetate supplementation. H proteins typically function as part of a glycine cleavage system in conjunction with T, P, and L proteins (5); however, there is no evidence of a P protein in malaria parasites (6, 7). Biochemical experiments indicate that malaria parasites do not perform glycine cleavage and knockout of the putative T protein in P. berghei demonstrates that this protein is not essential (30). By contrast, the H protein appears to be essential, since attempts to delete this protein in P. berghei were unsuccessful (30). These findings suggest that there is a noncanonical, essential role of the H protein in blood-stage malaria parasites. Whatever its function, it is unlikely that the H protein has any contribution to the synthesis of acetyl-CoA in the mitochondrion, and it is unlikely that acetate supplementation will bypass loss of H protein activity. Thus, H protein function may explain why acetate supplementation does not fully restore the growth of mS236A parasites and explain why we cannot generate parasites expressing fully active mLpa, even with acetate supplementation. This may also explain why parasites expressing the mKSA lipoamidase variant do not grow as well as wild-type parasites—the mKSA may be transiently binding to the lipoyl domain of all the lipoylated proteins in futile attempts to delipoylate them. In binding to the lipoyl domain of the H protein, the mKSA may be obstructing the H protein from performing some essential function merely by being in the way. Ultimately, if the H protein is essential for parasite growth, our results suggest that it must be lipoylated to perform its function.

In many replicate experiments, such as the one shown in Fig. 4B, lipoamidase was not able to completely cleave lipoate from PDH in the apicoplast. Initially, we thought that this could be a consequence of using the lower-strength RL2 promoter to drive the apicoplast constructs or that one of the two lipoylation domains found in PDH might be resistant to lipoamidase activity. Alternatively, factors associated with apicoplast trafficking such as the presence of the N-terminal apicoplast-targeting peptide or refolding of the trafficked protein could affect activity. All of these possibilities are discounted by the experiment shown in Fig. 4B which demonstrates that aLpa is able to rapidly cleave lipoate from PDH and the other lipoylated proteins when the organellar contents are mixed after cell lysis. This indicates that there is something specific about the apicoplast that limits lipoamidase activity. One possibility is that the conditions in the apicoplast, such as the pH and redox potential (31) or the presence of inhibitory compounds, could limit lipoamidase activity. If inhibitory compounds or conditions exist, their effects are reversed by cell lysis. Another possibility is that the LipB/LipA apicoplast lipoylation machinery simply works faster than lipoamidase activity. This would involve rapid action of the octanoyl transferase LipB, followed by addition of the lipoate sulfurs by the lipoate synthase LipA. It is hard to imagine rapid catalysis by LipA, since the protein donates sulfur atoms from its iron-sulfur cofactor to lipoate and must be repaired prior to each round of catalysis. Although it seems unlikely, apicoplast lipoylation remains a possibility in part due to uncertainty about how this pathway functions. Deletion of the LipB protein, which should be essential for apicoplast lipoylation, results in parasites that still have some residual PDH lipoylation in both P. falciparum and P. berghei (9, 32).

Our experiments demonstrate that reducing apicoplast lipoylation does not result in a discernible growth phenotype. This observation is consistent with knockout experiments of the apicoplast lipoylation machinery mentioned above and knockout of several subunits of the apicoplast PDH in several *Plasmodium* species (9, 23, 33, 34). Although PDH activity is essential for liver-stage malaria parasites (34), its activity in blood-stage parasites is puzzling. The apicoplast resident PDH should be fully competent to perform catalysis based on *in vitro* experiments (29), but it appears that it may not be performing this reaction at a detectable rate *in vivo* (23). Certainly, the substrate pyruvate is available in the apicoplast, since this is also a substrate for the essential isoprenoid biosynthesis pathway. If PDH activity is not needed for blood-stage parasites, then why is it produced and maintained in a lipoylated state?

Lipoamidase has proven to be an effective tool to probe *in vivo* lipoylation. The enzyme appears to be very specific for lipoylated protein and does not seem to affect any other important processes when expressed in either E. coli (13) or P. falciparum. Despite this substrate specificity, Lpa broadly recognizes all of the lipoylated proteins in these organisms, opening the door to the possibility of using this tool in other systems. The Lpa constructs generated in this project serve as a control for protein expression (Lpa versus KSA) and a means of probing cells with different levels of lipoamidase activity (Lpa versus attenuated mutants such as S236A). These constructs also demonstrate that Lpa can be tagged at the N terminus and C terminus, facilitating the use of organellar trafficking peptides and epitope tags. Adding this to inducible and knockdown systems available in other organisms offers a wide array of options to probe *in vivo* lipoylation, especially in systems in which the full array of lipoylation machinery has not yet been discovered. Our experiments with P. falciparum demonstrate that mitochondrial lipoylation is essential and implicate the BCDH and H protein as being responsible for this phenomenon. These results explain why lipoate scavenging is essential for parasite survival and imply that the lipoate attachment enzymes LipL1 and LipL2 will also be required for blood-stage parasite survival. Overall, these findings provide new insight into key aspects of mitochondrial metabolism in malaria parasites.

## MATERIALS AND METHODS

### Generation of the lipoamidase constructs in the pLZ E. coli expression vector.

Primers used for this work are listed in Table S1 in the supplemental material. We generated a series of lipoamidase constructs in the pLZ expression vector (4) (*malE* gene of pMAL_cHT [35] replaced with the amino acids MRGS). The pLZ vector allows us to evaluate the activity of any lipoamidase construct in E. coli as previously described (13). We began with pLZ plasmid pMS007, which contains the lipoamidase gene from Enterococcus faecalis strain V583 (13) and inserted a hemagglutinin (HA) tag by site-directed mutagenesis using the primers HA.F and HA.R, generating plasmid pMS011 encoding the construct Lpa. To generate Lpa containing a mitochondrial targeting sequence, the nucleotide sequence encoding the first 33 amino acids of the P. falciparum mitochondrial protein lipoate ligase 1 (LipL1, PF3D7_1314600) was amplified from P. falciparum cDNA using the primers L1_33_BamHI.F and L1_33_BamHI.R. This amplicon was inserted into the BamHI site at the 5′ end of the Lpa gene in plasmid pMS011 to create plasmid pMS030, which encodes the construct L1_33_Lpa. Apicoplast-targeted lipoamidase was produced by a similar method, except that the first 55 amino acids of the acyl carrier protein (ACP) (PF3D7_0208500) which encode the signal peptide and apicoplast transit peptide, were amplified from the plasmid pSP002 (36) using the primers ACP_55_BamHI.F and ACP_55_BamHI.R. Ligation of the digested amplicon into pMS011 produced the plasmid pMS021, containing the construct ACP_55_Lpa.

10.1128/mBio.01872-18.5 TABLE S1 Primers used to generate constructs and confirm integration. Codons modified through mutagenesis are shown in bold type, and endonuclease sites are underlined. Download Table S1, DOC file, 0.1 MB. Copyright © 2018 Jhun et al. 2018 Jhun et al. This content is distributed under the terms of the Creative Commons Attribution 4.0 International license.

Active site double mutants were engineered as controls for the active Lpa, although a single mutation of any of the catalytic triad residues to alanine was sufficient to inactivate the enzyme for *in vivo* studies in E. coli (13). The plasmids pMS011, pMS021, and pMS030 were mutagenized to contain the K159A/S259A double mutant by consecutive rounds of site-directed mutagenesis with primer sets K159A.F/K159A.R and S259A.F/S259A.R. The resulting plasmids, pMS015, pMS023, and pMS033, contain the double mutant constructs denoted KSA, ACP_55_KSA, and L1_33_KSA, respectively.

We generated a series of lipoamidase mutants based on the L1_33_Lpa construct with the goal of attenuating lipoamidase enzymatic activity. Site-directed mutagenesis was performed on pMS030 using primer pairs S236A.F/S236A.R, S236C.F/S236C/R, S236G.F/S236G.R, W210.F/W210.R, A256G.F/A256G.R, and Y375.F/Y375.R to produce plasmids pLZ-LpaS236A, pLZ-LpaS236C, pLZ-LpaS236G, pLZ-LpaW210F, pLZ-LpaA256G, and pLZ-LpaY375F, respectively. The rationale for making these mutations is shown in Fig. S2.

10.1128/mBio.01872-18.2 FIG S2 Structural depiction of lipoamidase mutagenesis rationale modeled from the X-ray crystal structure of 6-aminohexanoate cyclic dimer hydrolase (PDB 3A2Q). Mutations at the described positions were made to reduce lipoamidase enzymatic activity. These mutations were designed to disrupt carbonyl stabilization of the lipoamide bond (A256G), disrupt hydrogen bonding of the catalytic lysine (S236A/C/G), and interfere with hydrophobic interaction at the dithiolan ring of lipoate (W210F and Y375F). The catalytic triad (orange) is formed from K159, S235, and S259. Download FIG S2, TIF file, 2.5 MB. Copyright © 2018 Jhun et al. 2018 Jhun et al. This content is distributed under the terms of the Creative Commons Attribution 4.0 International license.

10.1128/mBio.01872-18.3 FIG S3 The mS236A lipoamidase variant is active, but not as efficient, as wild-type lipoamidase. Incubating the lysates of parasites expressing mS236A under nondenaturing conditions for 15 min (as in previous experiments) shows partial delipoylation of the PDH (reduction by 61.8%, SD = 19.3%, *n* = 5). However, extending this incubation to 1 h completely eliminates lipoylation signal. This evidence demonstrates that the mS236A variant has lipoamidase activity and suggests that it is less efficient than wild-type lipoamidase, a finding consistent with Fig. S1. Download FIG S3, TIF file, 1.6 MB. Copyright © 2018 Jhun et al. 2018 Jhun et al. This content is distributed under the terms of the Creative Commons Attribution 4.0 International license.

10.1128/mBio.01872-18.4 FIG S4 Acetate supplementation significantly improves growth in parasites expressing the mS236A lipoamidase variant. Growth curves from Fig. 6C transformed as relative growth of supplemented lines compared to their respective unsupplemented lines show that acetate supplementation rescues a growth defect that is statistically significant at days 4 and 6, with unsupplemented lines growing at 35.8% (SEM = 4.86%) and 36.2% (SEM = 11.6%), respectively (two-way ANOVA, followed by Bonferroni correction). Error bars indicate SEM. Download FIG S4, TIF file, 2.0 MB. Copyright © 2018 Jhun et al. 2018 Jhun et al. This content is distributed under the terms of the Creative Commons Attribution 4.0 International license.

### Evaluation of lipoamidase mutants with attenuated activity.

The pLZ constructs described above, as well as pMS030 and pMS033, were transformed into lipoylation-deficient JEG3 cells (19) in order to reduce background lipoylation signal in cell lysate experiments. Cells were induced with 0.4 mM isopropyl-beta-D-thiogalactopyranoside (IPTG) overnight at 20°C, and lysed in BugBuster (EMD Millipore), 1 mg/ml lysozyme, and 2.5 µg/ml DNase I for 10 min, and used immediately or frozen at −80°C. Lysates were analyzed by Western blotting for the presence of HA-tagged proteins to determine relative expression levels for each construct using ImageJ quantification. The lysates were then diluted in reaction buffer described below to normalize protein levels for comparative enzymatic assays.

The H protein from Bacillus subtilis was used as the substrate for the lipoamidase experiments. The coding region was amplified from genomic DNA using primers BsHprot.BamHI.F and BsHprot.SalI.R and cloned into the pMAL-cHT expression plasmid using BamHI and SalI. *E*. *coli* BL21-Star(DE3) cells (Invitrogen) were transformed with this plasmid, and the pRIL plasmid was isolated from BL21-CodonPlus-RIL cells (Agilent). Cells were induced at mid-log phase with 0.4 mM IPTG and harvested after growth at 20°C after 10 h. The H protein was purified by affinity chromatography and ion exchange using MBPTrap and HiTrap Q columns (GE Healthcare).

Cell-free lipoamidase activity assays were carried out using 6 µl of each cell lysate after normalization for lipoamidase concentration (as described above). Each reaction mixture was 60 µl in volume and contained final concentrations of 200 mM HEPES and 100 mM NaCl at pH 7.5 with 50 nM purified lipoylated H protein from Bacillus subtilis (serving as the substrate for the reaction). The reaction mixtures were incubated at room temperature for 30 min and were stopped by the addition of an equivalent volume of 6× Laemmli buffer. These samples were heated at 95°C for 4 min and vortexed for 1 min; this was performed twice for a total of 10 min. Lipoylated H protein was detected by Western blotting probed with 1:5,000 rabbit anti-lipoate antibodies (37) and 1:5,000 donkey anti-rabbit IgG horseradish peroxidase (HRP) secondary antibody (GE Healthcare). Quantitation of lipoylation signal was performed by ImageJ analysis. Results are summarized in Table S2 and Fig. S1.

10.1128/mBio.01872-18.6 TABLE S2 Coarse assessment of lipoamidase mutants. Using the same assay shown in Fig. S1, lipoamidase mutants were compared against WT lipoamidase activity. Protein levels were normalized by Western blotting (anti-HA) prior to the assay to ensure that cell lysates contained similar concentrations of each lipoamidase variant. Serial 10-fold dilutions were made for each variant and tested in a lipoamidase assay using the lipoylated H protein from B. subtilis as the substrate. Mutations at S236 appear to have been particularly effective at reducing activity, with the S236A mutations resulting in the slowest catalysis. Download Table S2, DOC file, 0.03 MB. Copyright © 2018 Jhun et al. 2018 Jhun et al. This content is distributed under the terms of the Creative Commons Attribution 4.0 International license.

### Construction of Plasmodium falciparum expression plasmids.

For expression in P. falciparum, lipoamidase constructs were cloned into the vector pLN-ENR-GFP (17) obtained from the MR4 (The Malaria Research and Reference Reagent Resource Center), or a derivative of this vector, pRL2 in which the strong calmodulin promoter was replaced with the weaker RL2 promoter (20). Lpa constructs were PCR amplified from the above-mentioned plasmid templates using the reverse primer HAAflII.R, and the following forward primers: LpaAvrII.F for plasmids pMS011 and pMS015, ACP_55_AvrII.F for plasmid pMS021, and L1_33_AvrII.F for plasmids pMS030 and pMS033. The resulting amplicons were digested with AvrII and AflII, followed by ligation into the same endonuclease sites in pLN-ENR-GFP. This produced six plasmids: pMS039 (encoding Lpa), pMS040 (encoding KSA), pMS026 (encoding ACP_55_Lpa), pMS036 (encoding L1_33_Lpa), pMS037 (encoding L1_33_KSA), and pLN-L1_33_S236A (encoding L1_33_S236A). Amplicons from plasmids pMS021 and pMS023 were also inserted into digested pRL2 to produce plasmids pMS049 and pMS050 which encode ACP_55_Lpa and ACP_55_KSA driven by the RL2 promoter.

### Parasite culture, transfection, and harvesting.

P. falciparum asexual blood-stage cultures were maintained at 2% hematocrit in complete medium consisting of RPMI 1640 medium with L-glutamine (US Biological Life Sciences) supplemented with 10% human serum or 5 g/liter AlbuMax II (Thermo Fisher), 20 mM HEPES, 23 mM NaHCO_3_, and 0.09 mM hypoxanthine. Transfections were performed as described previously (16); briefly, 400 µl of blood was washed with 5 ml of CytoMix and resuspended in 400 µl of CytoMix and 100 µg of the pINT plasmid encoding mycobacteriophage Bxb1 integrase (17) and 100 µg of the appropriate parasite expression vector. Drug selection was initiated 1 to 2 days after transfection. Where described, parasites lines were also supplemented with 5 mM sodium acetate. Growth curves were performed by seeding all genotypes at a starting parasitemia of 0.1%. Cultures were fed every other day, and parasitemia was determined on days 2, 4, and 6 by Giemsa-stained thin-film blood smears.

For Western blotting, autoradiography, and genomic DNA extraction, red blood cells from P. falciparum cultures were pelleted by centrifugation at 500 × *g* for 5 min before lysis of RBCs with 0.2% saponin for 3 min. The reaction was quenched with ice-cold PBS, and the parasites were pelleted by centrifugation at 3,000 × *g* for 10 min, followed by three washes with ice-cold PBS. The parasite pellets were used immediately or stored at −80°C.

### Immunofluorescence microscopy.

For mitochondrial staining, the parasite culture was treated with 75 nM MitoTracker Red CMX-Ros (Invitrogen) for 30 min in fresh media prior to fixation. All cultures were fixed in 4% paraformaldehyde–0.0075% glutaraldehyde on polylysine-treated slides for 30 min. Cells were permeabilized for 10 min in 1% Triton X-100, remaining aldehydes were reduced with 0.1 mg/ml NaBH_4_ for 10 min, and cells were blocked for 2 h in 3% BSA. Parasite cytosol was stained with 1:2,000 mouse anti-aldolase, the apicoplast was stained with 1:1,000 rabbit anti-ACP (38), and lipoamidase was stained with 1:500 rat anti-HA MAb 3F10 (Roche) overnight. After treatment with primary antibodies, the slides were washed and treated with cognate secondary antibodies. The cognate secondary antibodies were 1:2,000 rabbit α-mouse IgG Alexa Fluor 594 (rabbit anti-mouse IgG conjugated to Alexa Fluor 594 diluted 1:2,000), 1:2,000 goat α-rabbit IgG Alexa Fluor 594, or 1:2,000 goat αRat IgG Alexa Fluor 488 (Invitrogen). The slides were washed and mounted with Prolong Gold antifade reagent with DAPI (Invitrogen). Microscopy images were taken with the Zeiss Axio Imager. The PDM channel and PCC values were derived using Volocity software as previously described (39), and mean PCC values were obtained by analyzing multiple images from two independent experiments for each genotype.

### *In vivo* radiolabeling of mitochondrial substrates.

^35^S-labeled lipoate was prepared as previously described (4). Labeling with 0.2 μCi of ^35^S-radiolabeled lipoate was initiated in 1% hematocrit cultures with an initial parasitemia of 2%. Cultures were fed supplemented media, and blood films were made to monitor parasites every day for 48 h.

### Western blotting and autoradiography.

Parasite pellets as described above were resuspended in RIPA buffer (Boston Bioproducts) to determine lipoamidase activity in cell lysates, and the reaction was quenched by the addition of Laemmli loading buffer. Otherwise, pellets were resuspended in NuPAGE LDS sample buffer (Thermo Fisher) and alternatively vortexed for 1 min and boiled for 4 min, a total of two times. Proteins were resolved by SDS-PAGE on 4 to 12% gradient gels and transferred to nitrocellulose membranes. For autoradiography, membranes were dried overnight, and autoradiography was used to detect ^35^S-lipoate incorporation. For Western blotting, membranes were blocked and probed with 1:5,000 rat anti-HA MAb 3F10 (Roche) primary antibodies to detect lipoamidase, 1:5,000 rabbit antilipoate (37) to detect lipoylation of the apicoplast PDH E2 subunit, and either 1:10,000 mouse anti-HSP70 or 1:10,000 mouse antialdolase for loading controls.
