# Supplementary figures and images for: Using Lipoamidase as a Novel Probe To Interrogate the Importance of Lipoylation in Plasmodium falciparum
Source: mBio. 2018 Nov 20;9(6):e01872-18. doi: 10.1128/mBio.01872-18 (PMC6247088; doi:10.1128/mBio.01872-18)

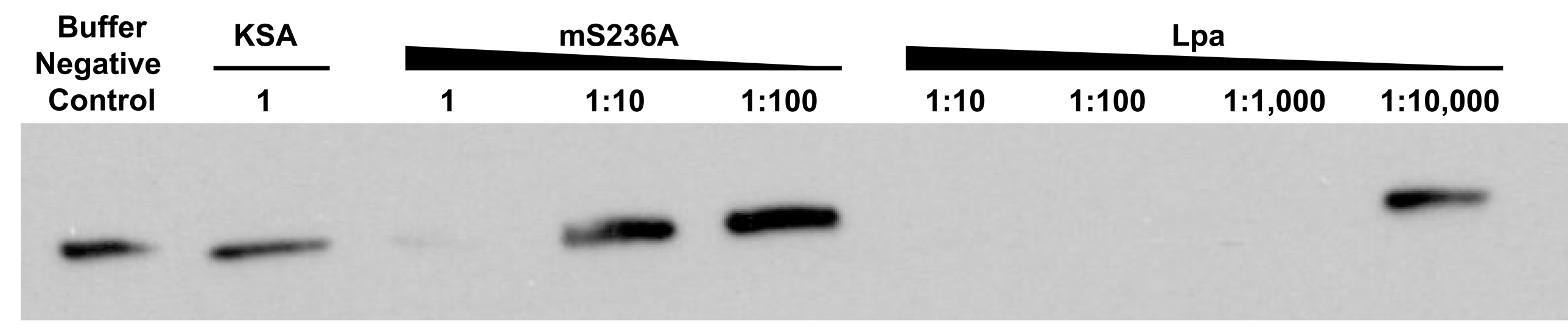

Supplement: FIG S1 [file mbo006184176sf1.tif]

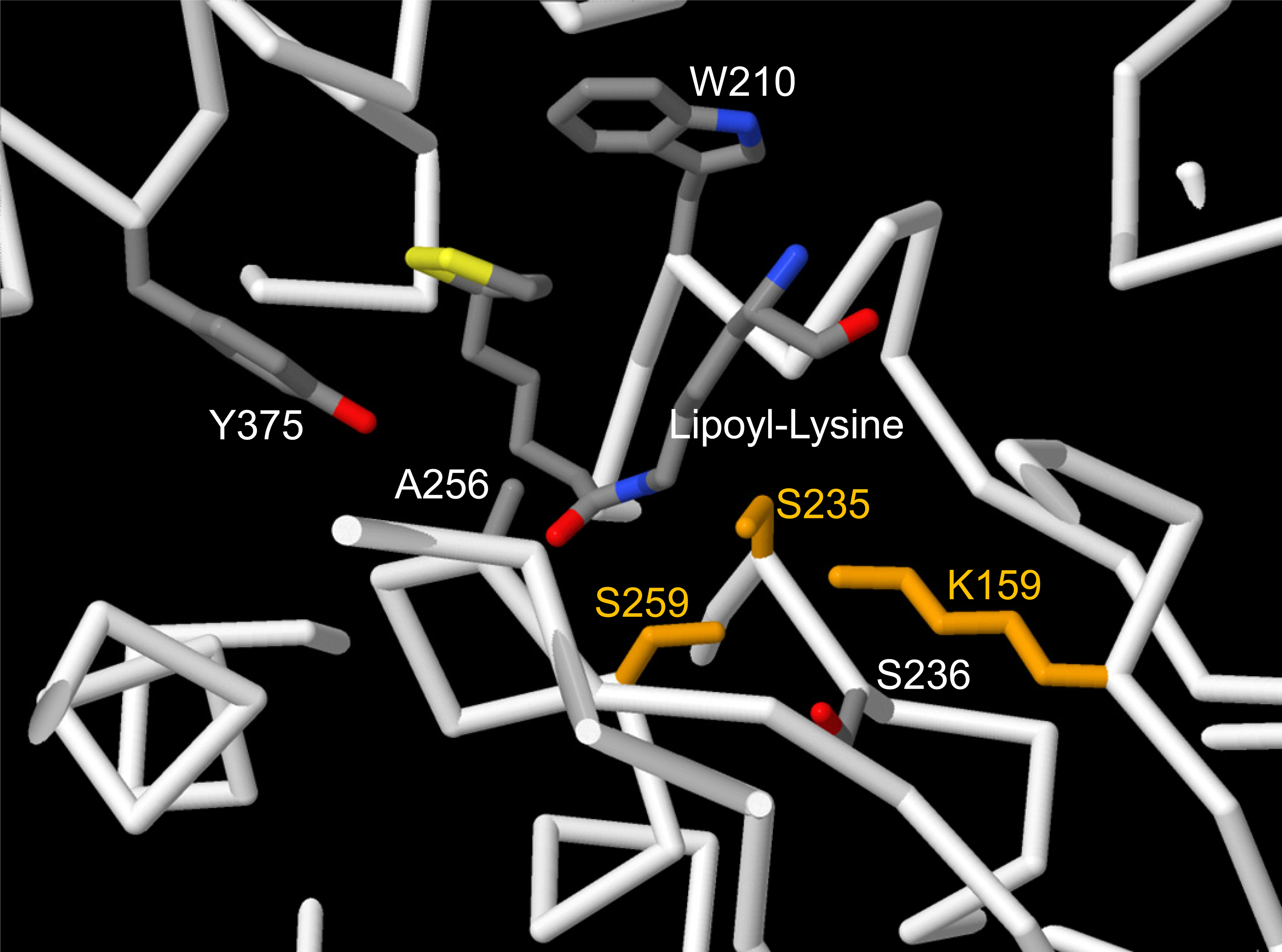

Supplement: FIG S2 [file mbo006184176sf2.tif]

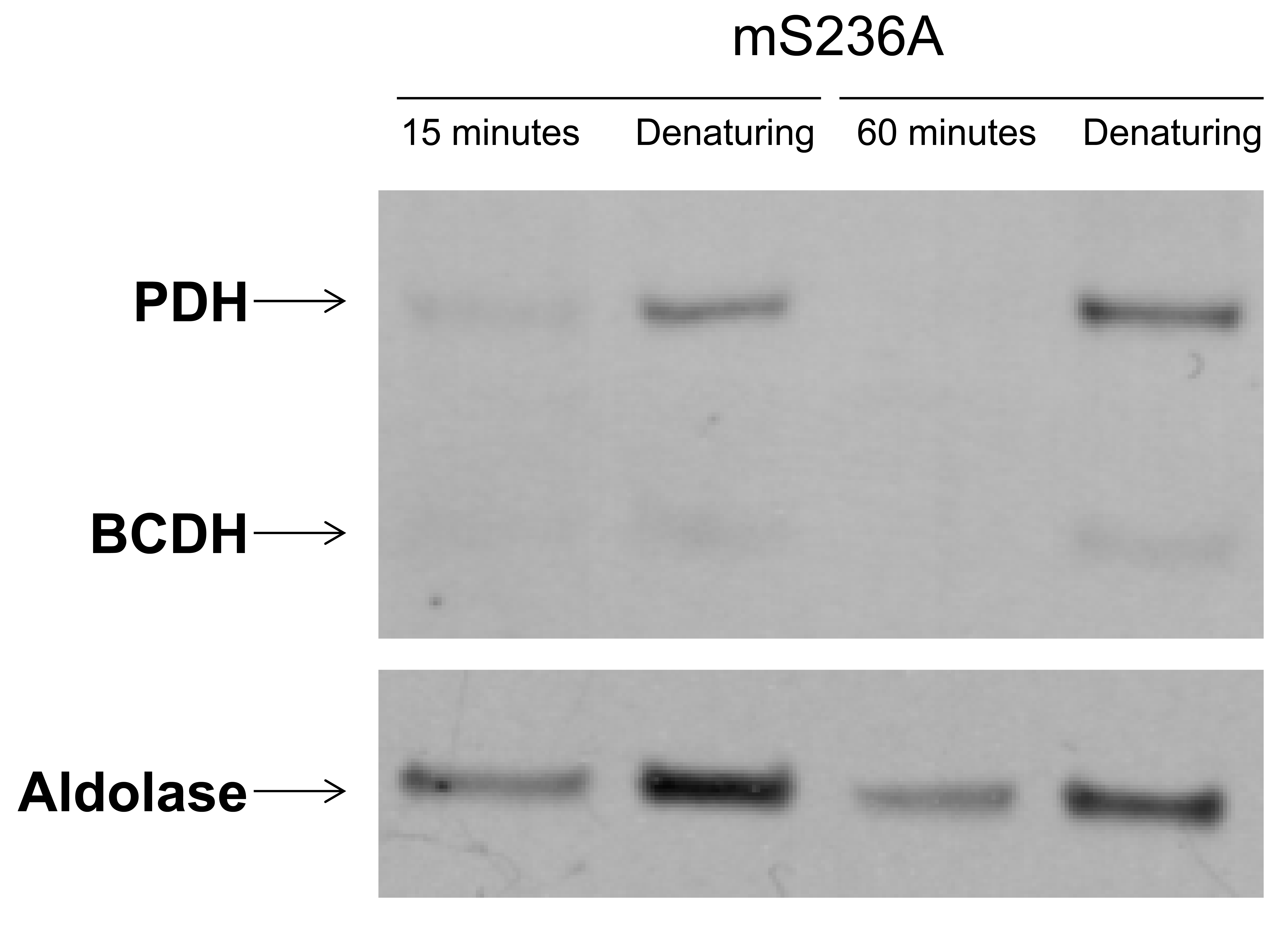

Supplement: FIG S3 [file mbo006184176sf3.tif]

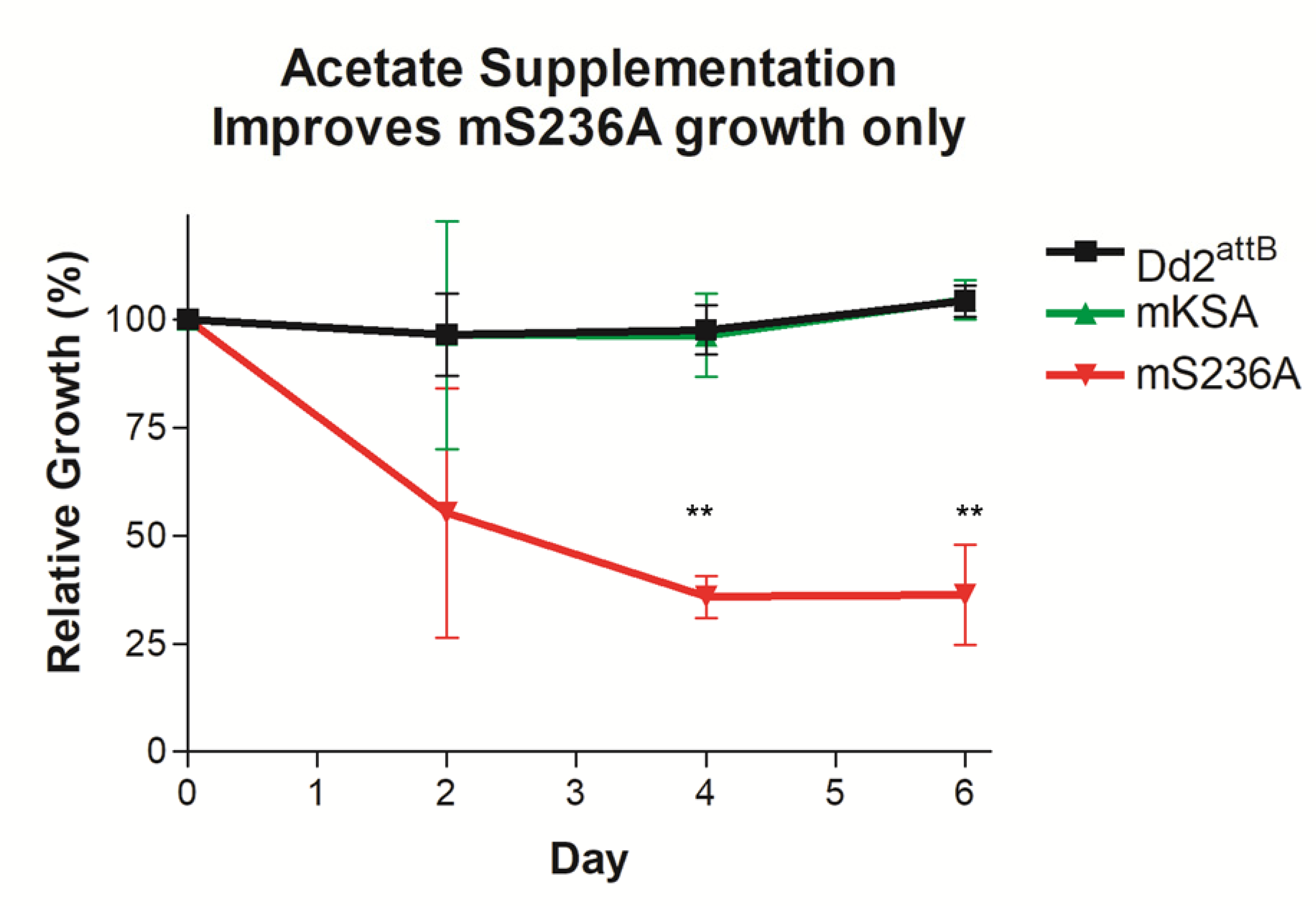

Supplement: FIG S4 [file mbo006184176sf4.tif]
