# Supplementary material for: Using Lipoamidase as a Novel Probe To Interrogate the Importance of Lipoylation in Plasmodium falciparum
Source: mBio. 2018 Nov 20;9(6):e01872-18. doi: 10.1128/mBio.01872-18 (PMC6247088; doi:10.1128/mBio.01872-18)
Supplement: TABLE S1 [file mbo006184176st1.doc]

| **Primer name** | **Sequence** |
| --- | --- |
| HA.F | GATTTATATAAGGAAAACTAGAAAA**TACCCATACGACGTCCCAGACTACGCT**TGATAAGTCGACCTGCAG |
| HA.R | CTGCAGGTCGACTTATCA**AGCGTAGTCTGGGACGTCGTATGGGTA**TTTTCTAGTTTTCCTTATATAAATC |
| L1_33_BamHI.F | GGTGGTGGATCCATGAAACGAATATTCAGGTTGGT |
| L1_33_BamHI.R | GGTGGTGGATCCATGAATATTCTGATTATTAGATACTAAT |
| ACP_55_BamHI.F | GGTGGTGGATCCATGAAGATCTTATTACTTTGTATAATTTTTC |
| ACP_55_BamHI.R | GGTGGTGGATCCTGGGTTTTTATTTTTTATCAAATTGTAATC |
| K159A.F | GGTGTGCCGCTCTTACTA**gcA**GGGTTAGGACAATCCTTG |
| K159A.R | CAAGGATTGTCCTAACCC**Tgc**TAGTAAGAGCGGCACACC |
| S259A.F | GAAGTGATGCTGGTGGC**gCT**ATCCGCATCCCTGC |
| S259A.R | GCAGGGATGCGGAT**AGc**GCCACCAGCATCACTTC |
| HAAflII.R | GGTGGTCTTAAGTCAAGCGTAGTCTGGGACGTCGTATGGG |
| LpaAvrII.F | GGTGGTCCTAGGATGttggcacaagaaagtatactag |
| ACP_55_AvrII.F | GTGGTCCTAGGATGAAACGAATATTCAGGTTGGTAAGA |
| L1_33_AvrII.F | GGTGGTCCTAGGATGAAGATCTTATTACTTTGTATAATTTTTC |
| InsKpnI.F | TGGTATTAGTATCTAATAATCAGAATATTCAT**GGTACC**ATGTTGGCACAAGAAAGTATACTAG |
| InsKpnI.R | CTAGTATACTTTCTTGTGCCAACAT**GGTACC**ATGAATATTCTGATTATTAGATACTAATACCA |
| FKBPKpnI.F | GGTGGTGGTACCATGGGAGTGCAGGTGGAAACCAT |
| FKBPKpnIR | GGTGGTGGTACCTTCCGGTTTTAGAAGCTCCACATC |
| S236A.F | GAATCCTAACCATTATTCAGGTGGTTCT**gCA**GGCGGAGCGGGTGCTAG |
| S236A.R | CTAGCACCCGCTCCGCC**TGc**AGAACCACCTGAATAATGGTTAGGATTC |
| S236C.F | GAATCCTAACCATTATTCAGGTGGTTCT**Tgt**GGCGGAGCGGGTGCTAGTG |
| S236C.R | CACTAGCACCCGCTCCGCC**acA**AGAACCACCTGAATAATGGTTAGGATTC |
| S236G.F | GAATCCTAACCATTATTCAGGTGGTTCT**ggA**GGCGGAGCGGGTGCTAGTG |
| S236G.R | CACTAGCACCCGCTCCGCC**Tcc**AGAACCACCTGAATAATGGTTAGGATTC |
| W210F.F | GGTCAAACGAATTATCCAGAATTAGGT**Ttt**AAGAATATTTCAGATTCAAAGTTATATGGCG |
| W210F.R | CGCCATATAACTTTGAATCTGAAATATTCTT**aaA**ACCTAATTCTGGATAATTCGTTTGACC |
| A256G.F | GTTCCCATTGCTTCTGGAAGTGAT**GgT**GGTGGCTCTATCCGCATCCCT |
| A256G.R | AGGGATGCGGATAGAGCCACC**AcC**ATCACTTCCAGAAGCAATGGGAAC |
| Y375F.F | GTTGATGGGGAACGTCTAATGAAAAAT**TtT**TATACTGTAGCTGCTGGCTCAGC |
| Y375F.R | GCTGAGCCAGCAGCTACAGTATA**AaA**ATTTTTCATTAGACGTTCCCCATCAAC |
| P1 | TGTATAAAAGATGAACATGGTGAATTC |
| P2 | GATAGCGATTTTTTTTACTGTCTG |
| P3 | GCACAGATGCGTAAGGAGAAAATACC |
| P4 | GCGCAATTAACCCTCACTAAAGGG |
| BsHprot.BamHI.F | TTTCAGGGCGGATCCATGAGCATACCAAAAGATTTGCGTT |
| BsHprot.SalI.R | GCCGCTCGAGTCGACTTAGTCTTCTTGTGTCATCTCTTCG |
