# Supplementary material for: Using Lipoamidase as a Novel Probe To Interrogate the Importance of Lipoylation in Plasmodium falciparum
Source: mBio. 2018 Nov 20;9(6):e01872-18. doi: 10.1128/mBio.01872-18 (PMC6247088; doi:10.1128/mBio.01872-18)
Supplement: TABLE S2 [file mbo006184176st2.doc]

| **Mutation** | **Approxmate Relative Catalysis to WT** |
| --- | --- |
| S236A | 1000X slower |
| S236C | 5X slower |
| S236G | 15X slower |
| Y375F | 10X slower |
| W210F | About the same as WT |
| A256G | About the same as WT |
